# Supplementary material for: Autoantibodies targeting GPCRs and RAS-related molecules associate with COVID-19 severity
Source: Nat Commun. 2022 Mar 9;13:1220. doi: 10.1038/s41467-022-28905-5 (PMC8907309; doi:10.1038/s41467-022-28905-5)
Supplement: Supplementary file 1 — Supplementary Information [file 41467_2022_28905_MOESM1_ESM.pdf]

Autoantibodies targeting GPCRs and RAS-related molecules associate with COVID-19 severity

Supplementary Figures 1-4

Supplementary Tables S1-S5 provided as Supplementary Data file

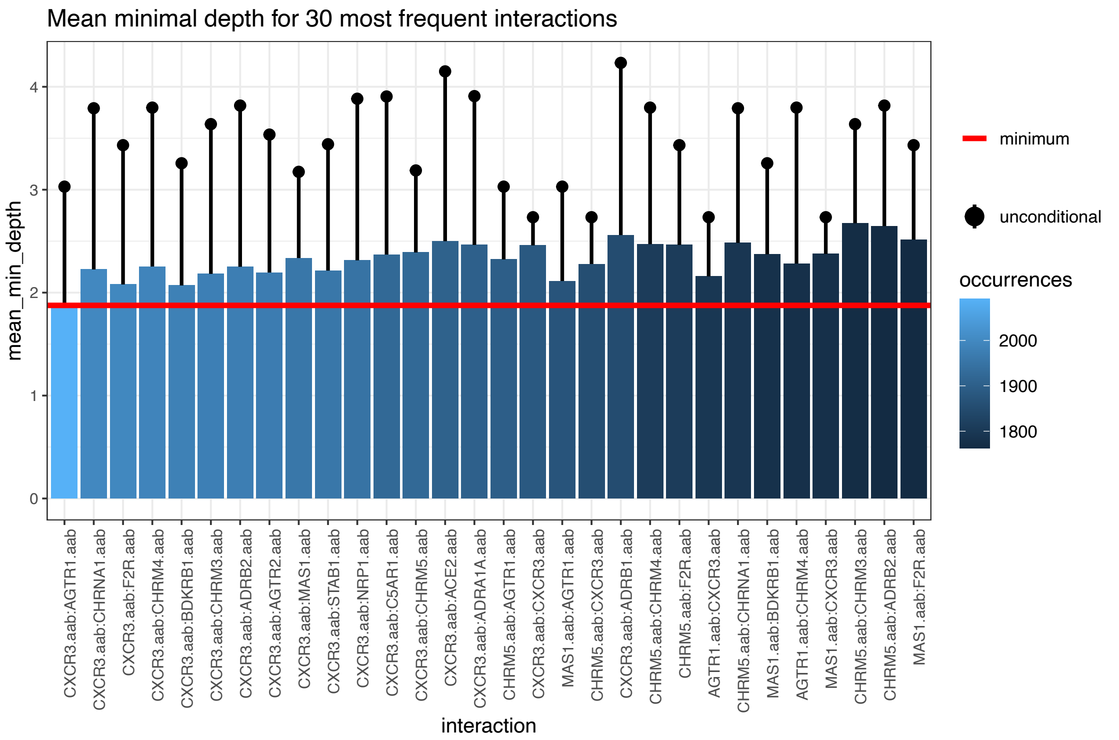

Supp. Fig. 1: Interactions between variables in the Random Forest. Interactions are ordered by decreasing number of occurrences. Thus, the plot shows, from left (lighter blue) to right (darker blue) the most frequently occurring interactions between the autoantibodies to the least frequently occurring interactions. The horizontal red line indicates the mean minimum depth, and the black lollipops indicate the unconditional mean minimal depth of the variables. Source data are provided as a Source Data file.

Supp. Fig 2

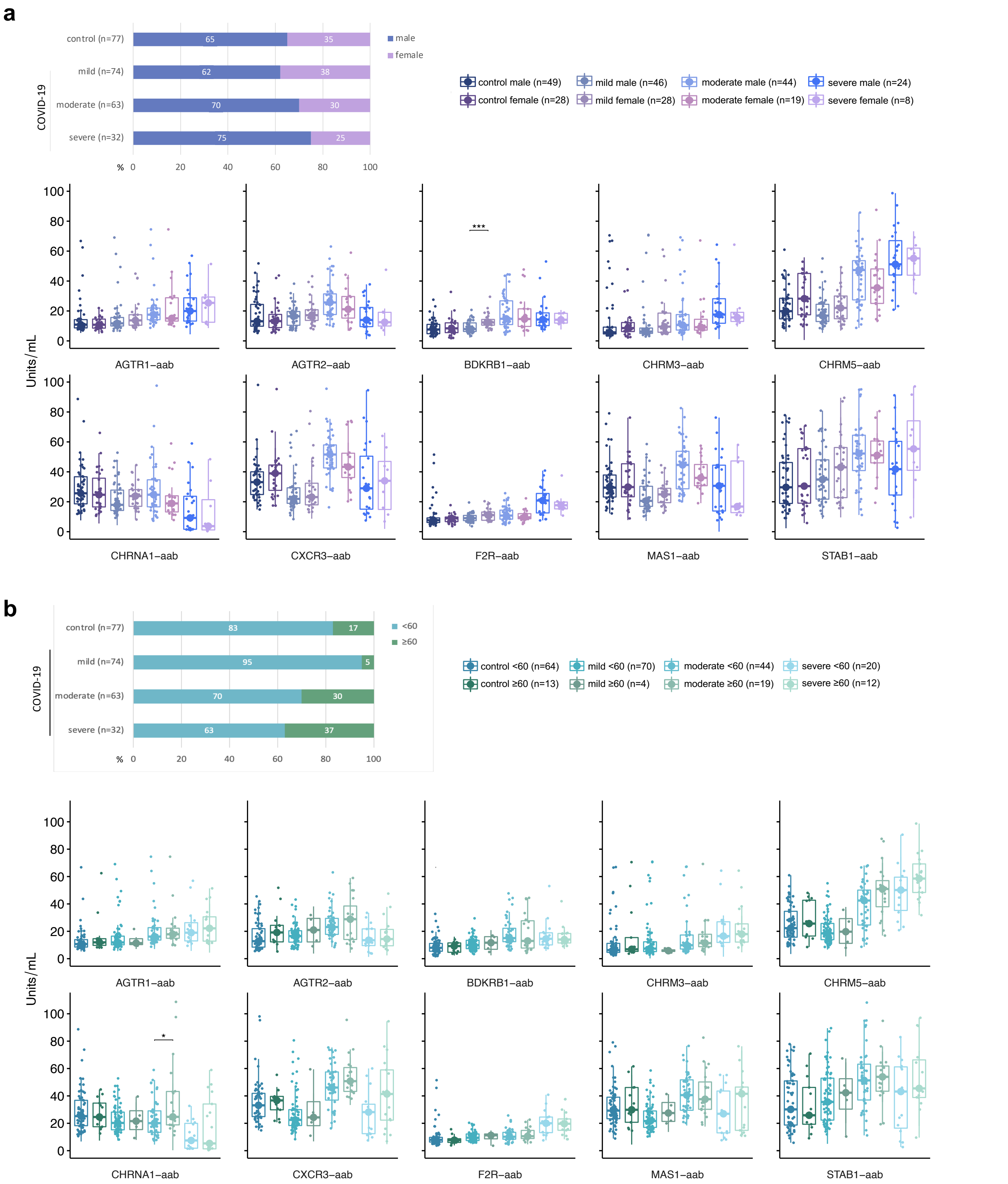

Supp. Fig. 2: Effect of sex and age on the levels of the top 10 autoantibodies ranked as predictors of disease severity by random forest analysis. Box plots of autoantibody levels of COVID-19 groups (mild, moderate or severe) versus healthy controls according to (**A**) sex and (**B**) age. Only intragroup comparisons were performed; that is, the group of control males was compared to the group of control females, mild COVID-19 males were compared with mild COVID-19 females, and so on. Each box plot shows the median with first and third interquartile range (IQR), whiskers representing minimum and maximum values within IQR, and individual data points. Significance was determined using two-sided Wilcoxon rank-sum tests and is indicated by asterisks (* $p \leq 0.05$, ** $p \leq 0.01$, *** $p \leq 0.001$, and **** $p \leq 0.0001$). Source data are provided as a Source Data file.

Supp. Fig 3

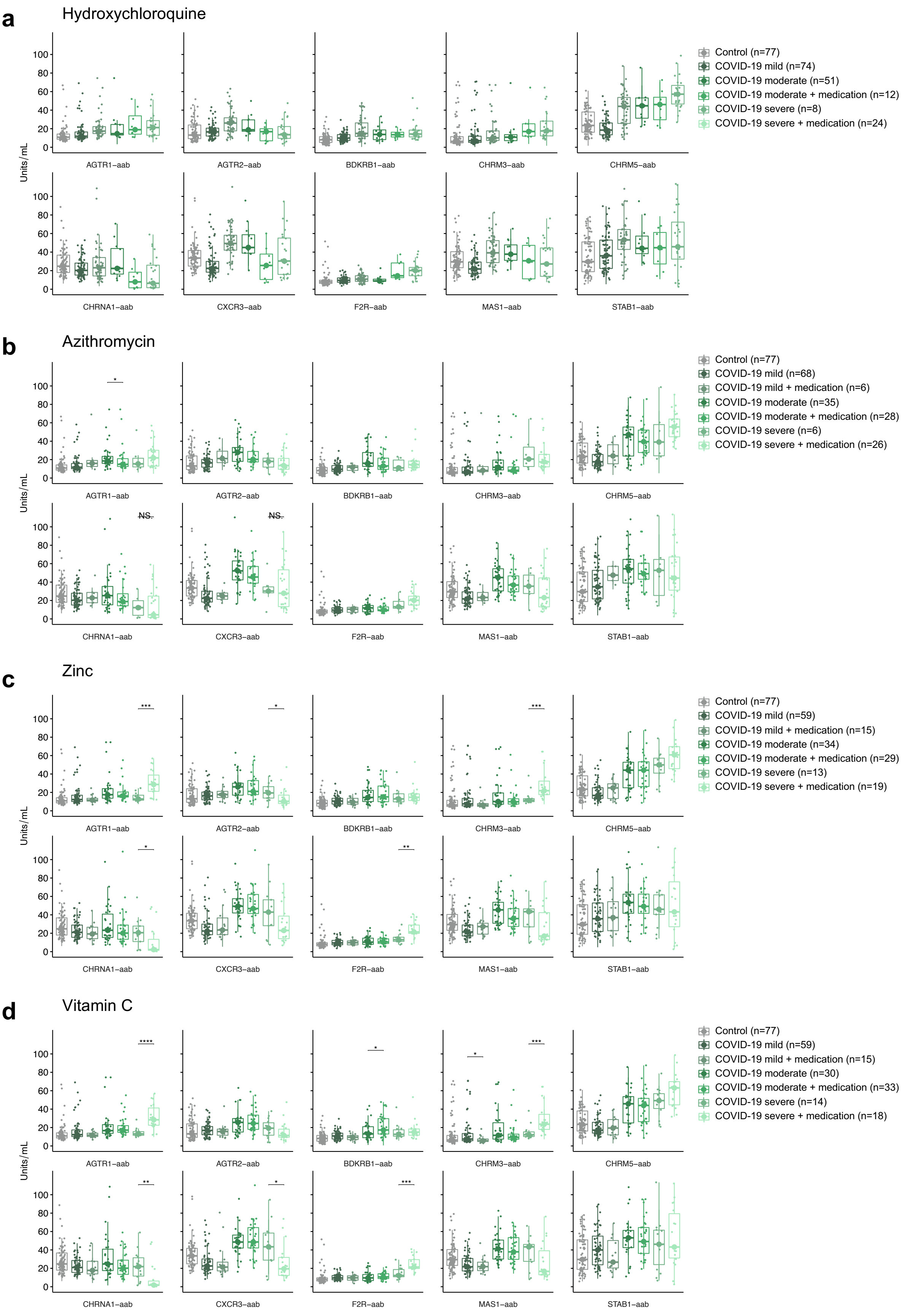

Supp. Fig. 3: Effect of treatments on the levels of the top 10 autoantibodies ranked as predictors of disease severity by random forest analysis. Box plots of autoantibodies investigated in COVID-19 groups (mild, moderate or severe) treated with or without (**A**) hydroxychloroquine, (**B**) azithromycin, (**C**) zinc, and (**D**) vitamin C. Only intragroup comparisons were performed to assess whether there were differences between mild, moderate, and severe COVID-19-treated versus untreated groups. There were no mild patients treated with hydroxychloroquine; thus, this group is not present in **A**. Each box plot shows the median with first and third interquartile range (IQR), whiskers representing minimum and maximum values within IQR, and individual data points. Significance was determined using two-sided Wilcoxon rank-sum tests and is indicated by asterisks (* p ≤ 0.05, ** p ≤ 0.01, *** p ≤ 0.001, and **** p ≤ 0.0001). Source data are provided as a Source Data file.

Supp. Fig 4

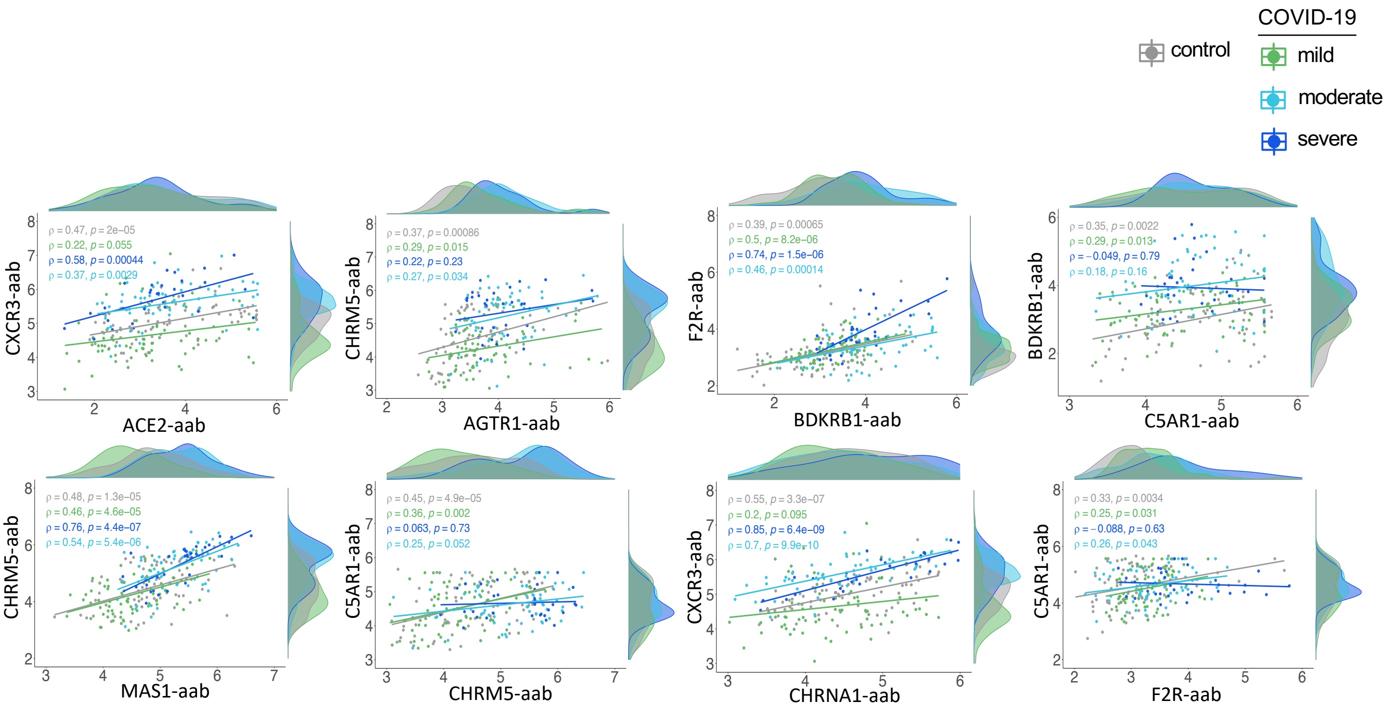

Supp. Fig. 4: Scatter plots with marginal density plots exhibiting the relationship between pairs of autoantibodies. Spearman's rank correlation coefficient (ρ) and significance level (*p*-value) for each correlation are shown within each graph. Histograms aside the graphics represent the density of sample (individual) distribution. Healthy controls n=77; COVID-19 groups: mild n=74, moderate n=63, and severe n=32. Source data are provided as a Source Data file.
