# Supplementary material for: Autoantibodies targeting GPCRs and RAS-related molecules associate with COVID-19 severity
Source: Nat Commun. 2022 Mar 9;13:1220. doi: 10.1038/s41467-022-28905-5 (PMC8907309; doi:10.1038/s41467-022-28905-5)
Supplement: Supplementary file 3 — Description of Additional Supplementary Files [file 41467_2022_28905_MOESM3_ESM.pdf]

Description of Additional Supplementary Files

File Name: Supplementary Data 1
Description: Autoantibody levels and metadata

File Name: Supplementary Data 2
Description: Log2-transformed autoantibody data.

File Name: Supplementary Data 3
Description: Autoantibody targets: gene names and functions.

File Name: Supplementary Data 4
Description: Gene ontology analysis of_autoantibody targets.

File Name: Supplementary Data 5
Description: Functional relationships between antibody targets and biological processes.
