# Supplementary material for: Autoantibodies targeting GPCRs and RAS-related molecules associate with COVID-19 severity
Source: Nat Commun. 2022 Mar 9;13:1220. doi: 10.1038/s41467-022-28905-5 (PMC8907309; doi:10.1038/s41467-022-28905-5)
Supplement: Supplementary file 9 — Reporting Summary [file 41467_2022_28905_MOESM9_ESM.pdf]

nature portfolio

Corresponding author(s): Otavio Cabral-Marques

Last updated by author(s): January 18, 2022

Reporting Summary

Nature Portfolio wishes to improve the reproducibility of the work that we publish. This form provides structure for consistency and transparency in reporting. For further information on Nature Portfolio policies, see our Editorial Policies and the Editorial Policy Checklist.

Statistics

For all statistical analyses, confirm that the following items are present in the figure legend, table legend, main text, or Methods section.

n/a	Confirmed	
☐	☒	The exact sample size (n) for each experimental group/condition, given as a discrete number and unit of measurement
☐	☒	A statement on whether measurements were taken from distinct samples or whether the same sample was measured repeatedly
☐	☒	The statistical test(s) used AND whether they are one- or two-sided<br>*Only common tests should be described solely by name; describe more complex techniques in the Methods section.*
☐	☒	A description of all covariates tested
☐	☒	A description of any assumptions or corrections, such as tests of normality and adjustment for multiple comparisons
☐	☒	A full description of the statistical parameters including central tendency (e.g. means) or other basic estimates (e.g. regression coefficient) AND variation (e.g. standard deviation) or associated estimates of uncertainty (e.g. confidence intervals)
☐	☒	For null hypothesis testing, the test statistic (e.g. F, t, r) with confidence intervals, effect sizes, degrees of freedom and P value noted<br>*Give P values as exact values whenever suitable.*
☒	☐	For Bayesian analysis, information on the choice of priors and Markov chain Monte Carlo settings
☐	☒	For hierarchical and complex designs, identification of the appropriate level for tests and full reporting of outcomes
☐	☒	Estimates of effect sizes (e.g. Cohen's d, Pearson's r), indicating how they were calculated

Our web collection on statistics for biologists contains articles on many of the points above.

Software and code

Policy information about availability of computer code

Data collection	Autoantibody levels were detected from frozen serum using commercial ELISA Kits (CellTrend, Germany) according to manufacturer’s instructions.
Data analysis	We used the R version 4.0.5 (The R Project for Statistical Computing. https://www.r-project.org/), R studio Version 1.4.1106 (R-Studio. https://www.rstudio.com/) for data analysis. R packages used to perform the data analysis include: ggpubr, lemon, ggplot2, ggExtra, prcomp vs princomp, randomForest (version 4.6.14), corrgram, psych, inlmisc , and CCA and whitening.<br>Gene ontology (GO) enrichment analysis of the 17 autoantibody targets was performed using GO Biological Process 2021 analysis through the Enrichr webtool (https://maayanlab.cloud/Enrichr/)<br>Circos Plot of antibody targets and pathway association was built using Circos online tool (http://www.circos.ca/software/).<br>IID ver 2021-0582 was used to search for physical protein interactions of the autoantibody targets, which was used to build a network figure prepared using NAViGaTOR version 3.0.1583. Final TIFF image with legends was prepared in Adobe Illustrator ver 26.0.

For manuscripts utilizing custom algorithms or software that are central to the research but not yet described in published literature, software must be made available to editors and reviewers. We strongly encourage code deposition in a community repository (e.g. GitHub). See the Nature Portfolio guidelines for submitting code & software for further information.

Data

Policy information about availability of data

All manuscripts must include a data availability statement. This statement should provide the following information, where applicable:

- Accession codes, unique identifiers, or web links for publicly available datasets
- A description of any restrictions on data availability
- For clinical datasets or third party data, please ensure that the statement adheres to our policy

The source data underlying the Main and Supplementary Figures are provided as a Source Data file.

Field-specific reporting

Please select the one below that is the best fit for your research. If you are not sure, read the appropriate sections before making your selection.

☒ Life sciences ☐ Behavioural & social sciences ☐ Ecological, evolutionary & environmental sciences

For a reference copy of the document with all sections, see nature.com/documents/nr-reporting-summary-flat.pdf

Life sciences study design

All studies must disclose on these points even when the disclosure is negative.

Sample size	We included 246 adults from Jewish communities across 5 states of the United States of America. Among them, there were healthy controls and patients who had developed symptomatic COVID-19 disease before receiving any SARS-CoV-2 vaccine.<br>Moreover, we worked with at least 30 samples by group, which is the double required to avoid spurious correlation results. Correlations on fewer than 15 samples will simply be too noisy for the network to be biologically meaningful. Thus, more samples normally lead to more robust and refined results.
Data exclusions	No data were excluded from the analyses
Replication	Autoantibody measurement have been replicated using commercial ELISA Kits (CellTrend, Germany).
Randomization	Individuals were grouped according disease state (Control and COVID-19).
Blinding	Investigators were blinded to group allocation during data collection. However, not to data analysis, where we need to determine patient groups to allow comparisons between disease outcomes and healthy controls.

Reporting for specific materials, systems and methods

We require information from authors about some types of materials, experimental systems and methods used in many studies. Here, indicate whether each material, system or method listed is relevant to your study. If you are not sure if a list item applies to your research, read the appropriate section before selecting a response.

Materials & experimental systems

n/a	Involved in the study	
☐	☒	Antibodies
☒	☐	Eukaryotic cell lines
☒	☐	Palaeontology and archaeology
☒	☐	Animals and other organisms
☐	☒	Human research participants
☒	☐	Clinical data
☒	☐	Dual use research of concern

Methods

n/a	Involved in the study	
☒	☐	ChIP-seq
☒	☐	Flow cytometry
☒	☐	MRI-based neuroimaging

Antibodies

Antibodies used	We have only measured autoantibodies detected using commercial ELISA Kits (CellTrend, Germany)
Validation	The ELISAs were validated according to the Food and Drug Administration's Guidance for Industry: Bioanalytical Method Validation.

Human research participants

Policy information about studies involving human research participants

Population characteristics	Details about the survey study, patient demographics and symptoms have been previously described (Ref 1 and 2). 77 randomly selected age- and sex-matched healthy controls (SARS-CoV-2 negative and without symptoms of COVID-19) were included in this study and their autoantibody data were compared to 169 individuals who were SARS-CoV-2 positive (determined by positive nasopharyngeal swab).<br><br>Ref 1) Silverberg, J. et al. Association of Varying Clinical Manifestations and Positive Anti-SARS-CoV-2 IgG Antibodies: A Cross-Sectional Observational Study. J. allergy Clin. Immunol. Pract. (2021) doi:10.1016/J.JAIP.2021.06.046.<br><br>Ref 2) Zyskind, I. et al. SARS-CoV-2 Seroprevalence and Symptom Onset in Culturally Linked Orthodox Jewish Communities Across Multiple Regions in the United States. JAMA Netw. open 4, (2021).
Recruitment	Participants were recruited as previously described:<br>Ref 1) Silverberg, J. et al. Association of Varying Clinical Manifestations and Positive Anti-SARS-CoV-2 IgG Antibodies: A Cross-Sectional Observational Study. J. allergy Clin. Immunol. Pract. (2021) doi:10.1016/J.JAIP.2021.06.046.<br><br>Ref 2) Zyskind, I. et al. SARS-CoV-2 Seroprevalence and Symptom Onset in Culturally Linked Orthodox Jewish Communities Across Multiple Regions in the United States. JAMA Netw. open 4, (2021).
Ethics oversight	All healthy controls and patients provided written consent to participate in the study, which was performed in accordance with the Declaration of Helsinki and approved by the IntegReview institutional review board. In addition, this study followed the Strengthening the Reporting of Observational Studies in Epidemiology (STROBE) reporting guideline.

Note that full information on the approval of the study protocol must also be provided in the manuscript.
